# The effect of off-center placement of twisted tape on flow and heat transfer characteristics in a circular tube

**DOI:** 10.1038/s41598-021-86285-0

**Published:** 2021-03-25

**Authors:** Li Wang, Peiyong Ni, Guannan Xi

**Affiliations:** School of Mechanical Engineering, Nantong University, Nantong, 226019 Jiangsu Province China

**Keywords:** Energy infrastructure, Energy infrastructure

## Abstract

This study is conducted to investigate the effect of off-center placement of twisted tape on flow distribution and heat transfer in a circular tube. The effect of tape width of 20, 18, 16, 14 and 12 mm on the heat transfer performance is discussed under the same twist ratio of 2.0. The numerical analysis of the flow field, average Nusselt number, friction factor and thermo-hydraulic performance parameter of the tube are discussed with Reynolds number ranged from 2600 to 8760. The results indicate that the Nusselt number of the tube fitted with center-placed twisted tapes at various width is 7–51% higher than the plain tube, and performance in low Reynolds region was found more effective than that in high Reynolds region. The heat transfer for circular tube with twisted tape attached to the wall shows better performance than that for the tube with center-placed twisted tape. With a smaller tape width, a higher increasing ratio of Nu-wall/Nu-center is obtained. The increasing ratio for Nusselt number ranged from 3 to 18%. However, the use of twisted tape inserts is not beneficial for energy saving. The thermo-hydraulic performance parameters for convective heat transfer of helium gas flowing in a circular tube are below unity for the calculated Reynolds region.

## Introduction

The need to reduce energy consumption has led to the development and usage of many heat transfer enhancement techniques. The enhancement in heat transfer also leads to the reduction of heat exchanger size and temperature driving force etc. In order to improve the efficiency of heat exchangers, heat transfer should be increased while avoiding excessive pump power. A variety of passive heat transfer enhancement techniques have been proposed, which do not consume additional energy other than that needed for circulating the working fluid. Detailed surveys have been made by Kareem et al.^1^, Grag et al.^2^, Rashidi et al.^3^ and some others. Passive techniques are applied widely to achieve higher energy efficiency, and examples include finned surfaces, twisted tape inserts, coiled tubes and so on. Among them, twisted tape can change the flow pattern in a channel, reduce thickness of the boundary layer and increase the heat transfer area. Many researches related to the effect of twisted tapes on heat transfer, pressure drop and friction factor in a circular tube have been carried out.

Manglik et al.^4,5^ conducted wide experiments to investigate heat transfer and pressure drop correlations for twisted-tape-inserts in isothermal tubes with Reynolds numbers ranged from laminar to transition and transient regions. Manglik et al.^6^ developed correlation for the isothermal Fanning friction factors for circular tubes with twisted-tape inserts. This correlation predicts experimental data for wide range of fluids, flow conditions and tape geometry to within 10%. Manglik et al.^7^ presented experimental flow visualization and computational modelling of single-phase laminar flows to clarify the mechanism of enhancement of heat transfer. Sharma et al.^8^ developed a combined approach to predict friction coefficients and heat transfer for a wide range of Re (200 < Re < 10^5^). Kurnia et al.^9^ numerically studied the flow behaviour and the heat transfer in helical tube with twisted tape insert. The flow in the tube is considered as laminar flow (Re = 100–2000). It is found that adding twisted tape insert in helical heat exchanger can enhance heat transfer performance by up to four times in comparison to conventional straight tubes.

Since the swirl flow generated by twisted tape decay in the free space between tape elements, some researchers considered to place twisted tapes with regular space in a tube or use increased tapered twisted tape. The use of regularly spaced twisted tape inserts will obviously decrease the pressure drop and lead to lower pump power compared with full-length twisted tape inserts. Saha et al.^10^ studied the heat transfer and pressure drop characteristics in circular tube fitted with regularly spaced twisted-tape elements which were connected by thin circular rods. This study aims at twisted-tape induced laminar tube flow with uniform heat flux and the effect of twist ratio, space ratio, tape-width, rod-diameter and phase angle between tape elements were investigated. It is concluded that the increase of phase angle is of no use, instead it increases the manufacturing complexity. Eiamsa-ard et al.^11^ studied the heat transfer enhancement and pressure loss by insertion of single twisted tape, full-length dual and regularly-spaced dual twisted tapes in a round tube under axially uniform wall heat flux conditions. This comparative investigation shows that the heat transfer of the tube with dual twisted tapes is higher than those with single twisted tape insert, and all dual twisted tapes with free spacing yield lower heat transfer enhancement in comparison with the full-length dual twisted tapes. Eiamsa-ard et al.^12^ presented a case study on thermal performance of a heat exchanger tube equipped with regularly spaced twisted tapes. A numerical simulation was conducted to show flow structure, temperature field and local Nusselt number. The RNG k–ε turbulence model was used and the predicted values of Nusselt number, friction factor and thermal performance factor are respectively within ± 5%, ± 7% and ± 3% of the experimental data. Fagr et al.^13^ and Mushatet et al.^14^ studied the insertion of decreased tapered twisted tape and increased tapered twisted tape inside a circular tube, respectively. Both experimental and numerical study were conducted and the RNG k–ε model was chosen as turbulence model. According to their research, there is no significant difference in thermal performance factor between some studied cases of inserting decreased tapered twisted tape and the case of inserting typical twisted tape. In contrast, for increased tapered twisted tape, the decrease in the starting width leads to an increase in the Nusselt number as a result of the increase in the angular momentum.

Various modified twisted tapes have been studied and some was proved to show better heat transfer, friction factor and thermal enhancement factor than plain twisted tapes. Murugensan et al.^15^ carried out experimental study for a double pipe heat exchanger equipped with square-cut twisted tapes and plain twisted tapes. The results showed that better performance of heat transfer, friction factor and thermal enhancement factor was obtained by using square-cut twisted tape, which is due to the higher generation of additional disturbance and secondary flow at vicinity of tube wall. Kumar et al.^16^ studied the spiral plate heat exchanger by computational analysis method. The flow pattern and heat transfer are analysed. Chandrasekar et al.^17^ studied the heat transfer and pressure drop in double helically coiled tube heat exchanger with nanofluids, the effect of volume concentration of the nanofluid was discussed. Liu et al.^18^ numerically studied the heat transfer enhancement characteristics of coaxial cross twisted tapes for tube flows in laminar region. The Nusselt number of the tube inserted with coaxial cross double twisted tape (CCDTT) and coaxial cross triple twisted tape (CCTTT) were compared to that inserted with traditional twisted tape (TTT). The result shows that with CCTTT as insert, the Nusselt number is 1.51–1.95 times of that the tube equipped with TTT, and is 1–27% higher than that of the CCDTT for Re ranged from 40 to 1050. Abolarin et al.^19^ investigated the heat transfer and pressure drop in a circular tube with alternating clockwise and counter clockwise twisted tape inserts. The Reynolds number ranged from 300 to 11,404 covering laminar, transitional and turbulent flow regimes. The effect of connection angle was discussed and empirical correlations were developed.

The premise of most researches mentioned above is that the width of the twisted tape is equal or almost equal to the tube diameter and the twisted tape is placed along the center axis of the tube. To facilitate assembly, disassembly and eliminating dirt, twisted tapes are usually designed with a width smaller than the tube diameter. Some researchers have studied the effect of width of the twisted tape and some even tried to place several twisted tapes with small width in the tube. Al-Fahed and Chakroun^20^ experimentally studied the clearance between the tube and equipped twisted tapes and it is concluded that the heat transfer increases with the decrease of the clearance. Bhuiya et al.^21^ studied the heat transfer augmentation in a circular tube with perforated double counter twisted tape inserts. According to this research, the heat transfer rate and friction factor were about 80–290% and 111–335% higher than those of the plain tube, respectively.

So far in the literature, twisted plates are generally designed to be placed along the center axis of the circular tube and is coaxial with the tube. However, in actual operating conditions, when the width of twisted tape is smaller than the tube diameter, twisted tape is not precisely maintained at the centre axis of the tube. Therefore, it is worthwhile studying the offset effect of twisted tape, which could serve as a guideline to the design and usage of this kind of heat transfer equipment.

In the present study, we attempt to figure out the effect of off-center placement of twisted tape on flow distribution and heat transfer in a circular tube. Twisted tapes with different width are offset to contact with the tube wall. The flow field, average Nusselt number, friction factor and thermo-hydraulic performance parameter of the tube are discussed with different placement of twisted tape. The effect of heat transfer enhancement induced by twisted tape inserts is discussed.

## Geometric structure and simulation model

### Physical model

**Figure 1:**
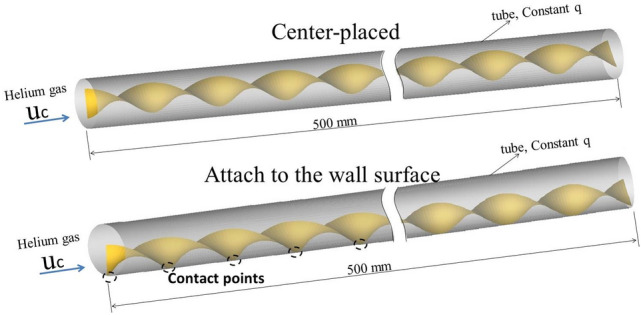
A schematic diagram for the tube with center-placed and attach to wall surface twisted tape (W = 12 mm).

**Figure 2:**
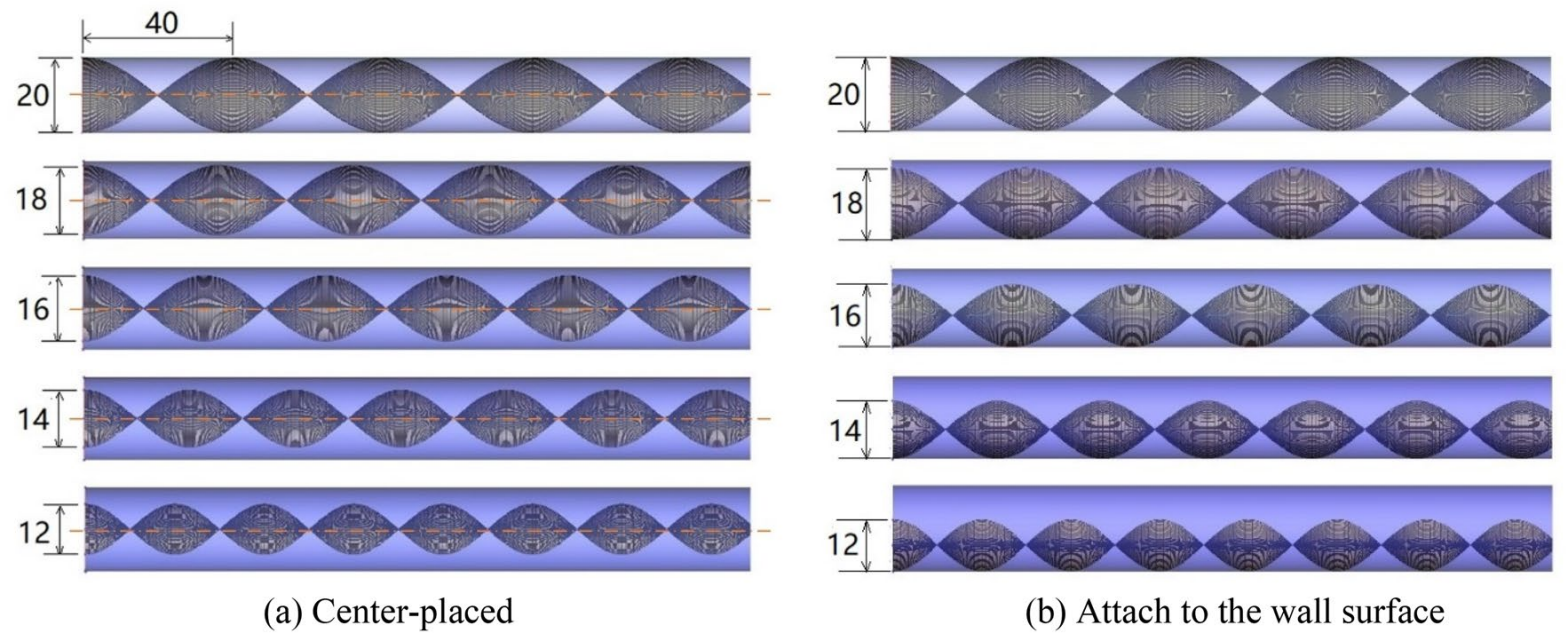
Twisted tape inserts with different width.

A full dimension schematic diagram was shown in Fig. 1. The width (W) of the twisted tape is 12 mm. The twisted ratio (s = H/W) is set as 2.0. The inner diameter (D) of the tube is 20 mm and the thickness 1 mm. The total length of the tube is 500 mm. The geometries of the circular tube with center-placed twisted tape and off-center placed (attach to the tube wall) twisted tape are shown in Fig. 2a,b, respectively. Twisted tapes with thickness of 0.1 mm and width (W) of 20, 18, 16, 14 and 12 mm are used as inserts for the circular tube, and thus the relative 180° twisted pitches are 40, 36, 32, 28 and 24 mm, respectively.

### Governing equations and boundary conditions

Helium gas is selected as the working fluid with incompressible assumption. The thermo-physical properties of helium gas, such as thermal conductivity, density, and dynamic viscosity are dependent on temperature, as shown in Table 1. The flow inside the tube is assumed to be steady and natural convection is neglected. The problem is considered as a three-dimensional turbulent heat transfer problem in steady state. Heat conduction in the twisted tape is neglected. The governing equations of continuity, momentum and energy for helium gas are given below in a tensor form.

1 $$\frac{\partial {u}_{i}}{\partial {x}_{i}}=0$$

2 $$ \frac{{\partial \left( {u_{i} u_{j} } \right)}}{{\partial x_{j} }} = - \frac{1}{\rho }\frac{\partial p}{{\partial x_{i} }} + \nu \frac{{\partial^{2} u_{i} }}{{\partial x_{j}^{2} }} - \frac{\partial }{{\partial x_{j} }}\left( {\overline{{u_{i}^{\prime } u_{j}^{\prime } }} } \right) $$

3 $$ \frac{\partial }{{\partial x_{i} }}\left( {\rho u_{i} T} \right) = \frac{\partial }{{\partial x_{j} }}\left[ {\left( {\frac{k}{{c_{P} }} + \frac{{\mu_{t} }}{{Pr_{t} }}} \right)\frac{\partial T}{{\partial x_{j} }}} \right] $$

**Table 1:** Thermo-physical properties of helium gas^22^.

Fluid	Property	Temperature range (K)	Value or correlation
Helium gas	Thermal conductivity, *k* (W/m K)	255.6–500	3.94 + 3.59 × 10^−3^ T − 1.98 × 10^−9^T^2^ + 3.19 × 10^−12^T^3^ − 9.77 × 10^−16^T^4^
	Density, *ρ* (kg/m^3^)	300–500	P/RgT
	Specific heat, *c*_*p*_ (J/kg K)	300–500	5197
	Thermal conductivity, *k* (W/m K)	300–500	1.034 × 10^–1^ + 2.58 × 10^-4^ T
	Dynamic viscosity, *μ* (Pa s)	300–500	1.307 × 10^–5^ + 3.319 × 10^−8^ T
	Prandtl number, Pr	300–500	0.68

The Reynolds number (*Re*), the Reynolds number considering the helically velocity (*Re*_*s*_), the convective heat transfer coefficient of the tube flow (*h*), the Nusselt number (*Nu*), and the friction factor (*f*) are defined as follows:

4 $$ \begin{gathered} Re = \frac{{\rho u_{c} D}}{\mu } \hfill \\ Re_{s} = \frac{{\rho u_{s} D}}{\mu } \hfill \\ \end{gathered} $$

5 $${u}_{s}={u}_{c}{[1+{(\pi /2s)}^{2}]}^{0.5}$$

6 $$ h = \frac{{\rho Du_{c} C_{P} \left( {T_{in} - T_{out} } \right)}}{{4L\left[ {\frac{{T_{in} + T_{out} }}{2} - T_{W} } \right]}} $$

7 $$Nu=\frac{hD}{{k}_{f}}$$

8 $$f=\frac{\Delta P}{(\rho {u}_{c}^{2}/2)(L/D)}$$

where $D$ is the inner diameter of the tube, m.

The average velocity of inlet varies from 4 to 12 m/s, with corresponding Reynolds number ranged from 2600 to 8760. Taking the swirl effect into consideration, the swirl Reynolds number (*Re*_*s*_) ranged from 3300 to 11,140. Therefore, the flow is in turbulent zone. In this study, the value of $Re$ is used for the results and discussions. Reynolds averaged Navier–Stokes (RANS) turbulence models of RNG k–ε Model (RNG) as well as the Reynolds Stress Model (RSM) are adopted with enhanced wall treatment for near wall modelling, and the simulation results are compared in this study. The exit condition is set to pressure outlet, P = 500 kPa.

Non-slip boundary conditions are used for the twisted tape surface and the inner wall of the channel. The boundary condition between helium gas and the surface of twisted tape is fluid–structure interaction. Constant heat flux boundary condition is loaded on the outer surface of the tube. The CFD software ANSYS (version 14.0) is used for numerical computation based on the finite volume method. The SIMPLE algorithm was used and the convergence criterion is set 10^–4^ for residuals of the continuity equation, and 10^–6^ for the momentum and energy equations.

Boundary conditions: 

at the twisted tape surface,

9 $$u=v=w=0$$

10 $$ \left. {T_{s} } \right|_{solid} = \left. {T_{s} } \right|_{fluid} $$

at inner wall of the channel,

11 $$u=v=w=0$$

12 $$-\lambda {\left.\frac{\partial T}{\partial Z}\right|}_{z=0}=q$$

where, *λ* (W/(m K)) is the thermal conductivity.

### Grid independent analysis

A grid independent test was performed before the numerical analysis. Hexahedral dominant grid is used for the mesh and the grid in the region near the tube wall and the twisted tape surface are highly refined to ensure the mesh quality for boundary layer resolution. The stabilization of the calculated results of the surface temperature of the tube wall (T_w_), the outlet temperature (T_out_) and the friction factor (f) are taken as evaluation parameters for the gird independence. Four grid systems with different average grid size are used to calculate the same case. The twisted tape with width of 20 mm is placed coaxial with the circular tube. The twist ratio is 2.0 and the relative 180° twisted pitch is 40 mm. The inner diameter of the tube is 20 mm and the total length is 500 mm. The inlet velocity is 10 m/s and the outlet pressure is set to 500 kPa. The T_w_, T_out_ and f are shown in Table 2.

**Table 2:** Grid independence test.

Model	Grid number	Tw (K)	Tout (K)	f
Model-I	910,000	368.29	351.65	0.1565
Model-II	1,700,000	370.94	351.97	0.1566
Model-III	2,839,000	372.11	352.09	0.1563
Model-IV	4,270,000	372.24	352.16	0.1489

It can be found that the *T*_*w*_ increases with the increase of grid number. The Model-I and Model-II are too coarse with the differences of 1.1% and 0.35% when compared to Model-IV. The difference for *T*_*w*_ between model-III and model-IV is very small (less than 0.1%). The result of *T*_*out*_ changes very little for the four grid systems, the maximum difference is 0.14%. The friction factor calculated by Model-I has a rather large difference of 4.9% compared to Model-IV, while the differences between the other three models are very limited (less than 0.2%). To balance the grid accuracy and the computing cost, the basic grid size of Model-III is chosen as the grid system for all calculations in this study.

### Verification of numerical models

**Figure 3:**
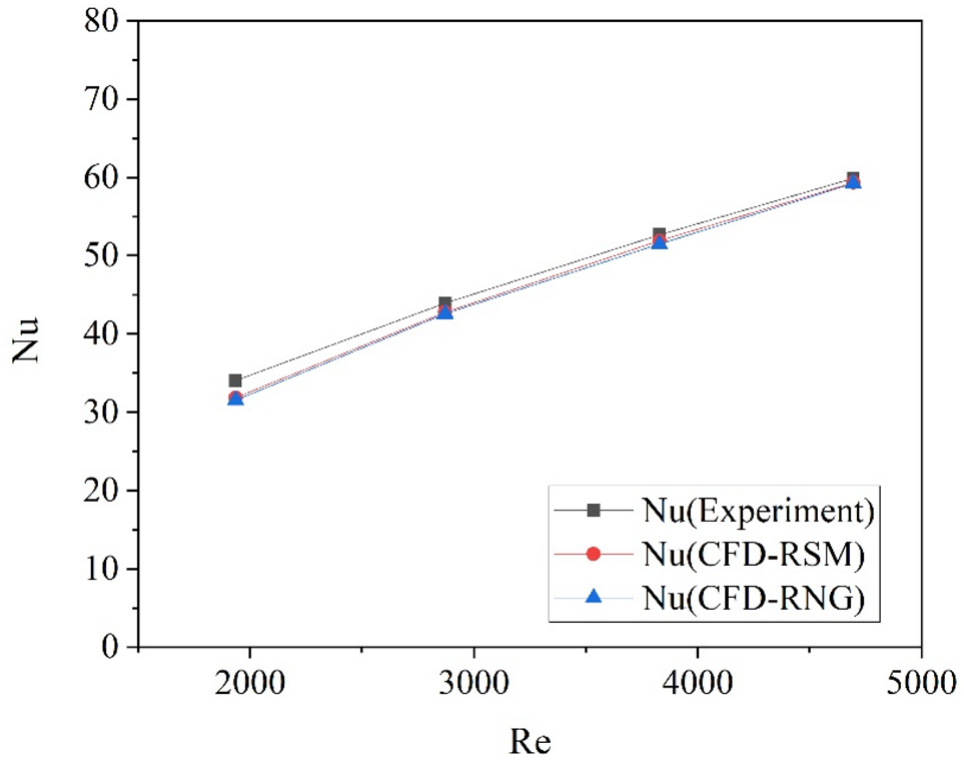
Comparison for the calculated Nusselt numbers of twisted tape with experimental data.

In our previous experimental research^23^, heat transfer coefficients for helium gas flowing over a twisted tape was obtained. The width of the tape is 4 mm and the relative 180° twisted pitch is 20 mm with corresponding twist ratio of 5. The twisted tape was put along the center axis of the tube which has inner diameter of 20 mm. The inlet velocity of helium gas ranged from 4 to 10 m/s and the inlet pressure is maintained at 500 kPa. A numerical simulation case was built with the same conditions as the experiment to validate the flow and heat transfer process over the twisted plate. Turbulence models of RNG and the RSM are used, and the simulation results for *Nu* was compared with experimental data at various Reynolds numbers. As shown in Fig. 3, deviation between the numerical results and experimental data is very limited. The maximum error occurs at the lowest Reynolds number, and is of 6.4% for the RSM model and 7.6% for the RNG model, respectively. This demonstrates that the accuracy of the numerical models is acceptable, and the RSM model shows better performance. Therefore, the RSM turbulence model is applied for the following simulations.

The above validation was for the Nusselt number of the twisted tape which is put in a circular tube. Since heat transfer over the twisted tape is strongly associated with the flow field around the twisted tape, the accuracy of heat transfer prediction can also reflect the accuracy of flow field calculation to some extent.

**Figure 4:**
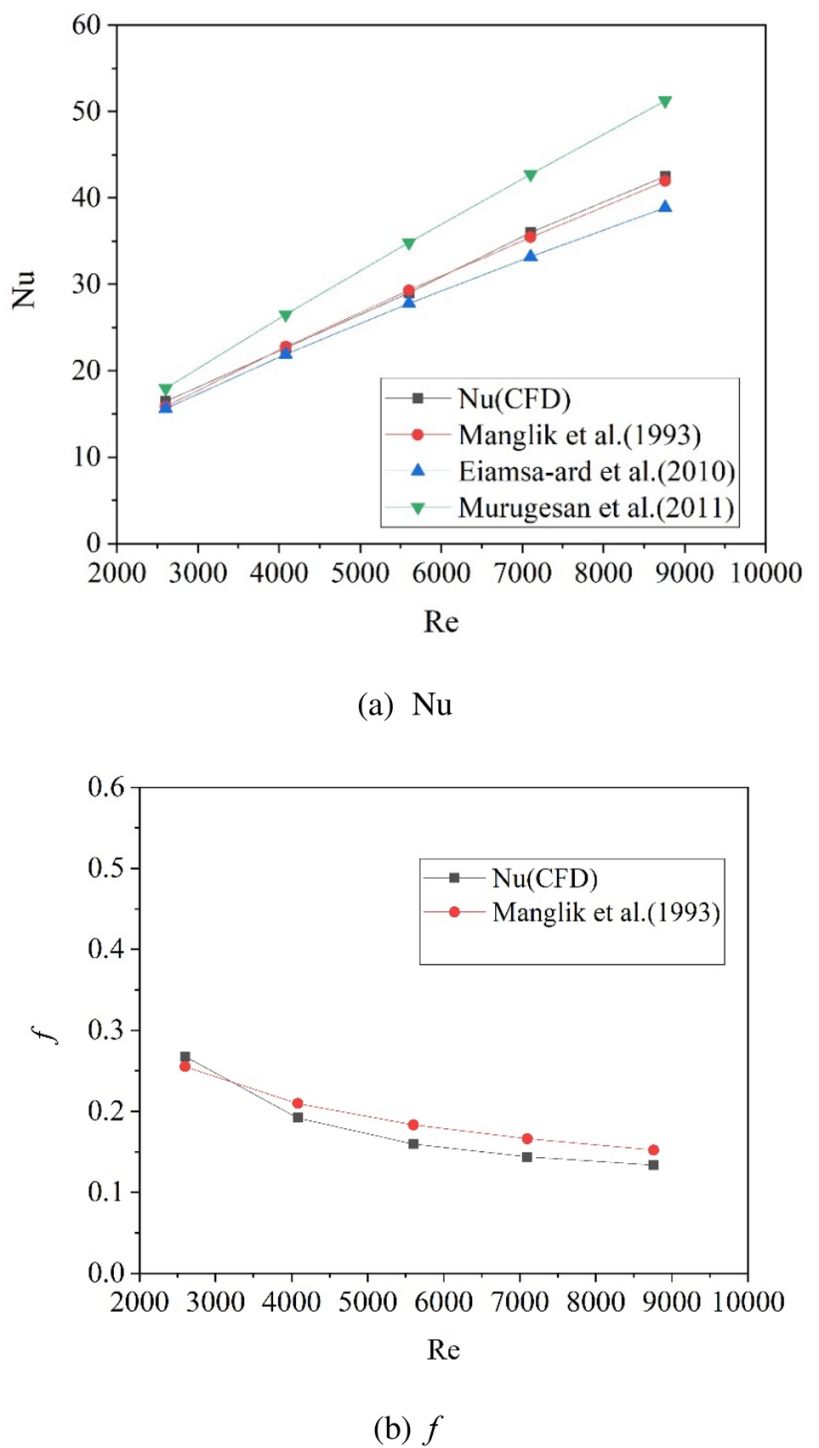
Comparison of the calculated *Nu* and *f* with empirical correlations for the tube with twisted inserts.

As for the heat transfer of the circular tube, numerical results of the *Nu* and *f* calculated for Model-III in “Grid independent analysis” were compared with empirical correlations proposed by Manglik et al.^4,5^, Eiamsa-ard et al.^11^ and Murugesan et al.^15^, as shown in Fig. 4. The Nusselt numbers calculated by CFD method matches the correlation proposed by Manglik et al. best, and the overall deviations of the calculated *Nu* and *f* from the empirical correlations are found to be within 3.8% and 13.4%, respectively. Therefore, the accuracy of the numerical method is validated.

## Flow characteristics

**Figure 5:**
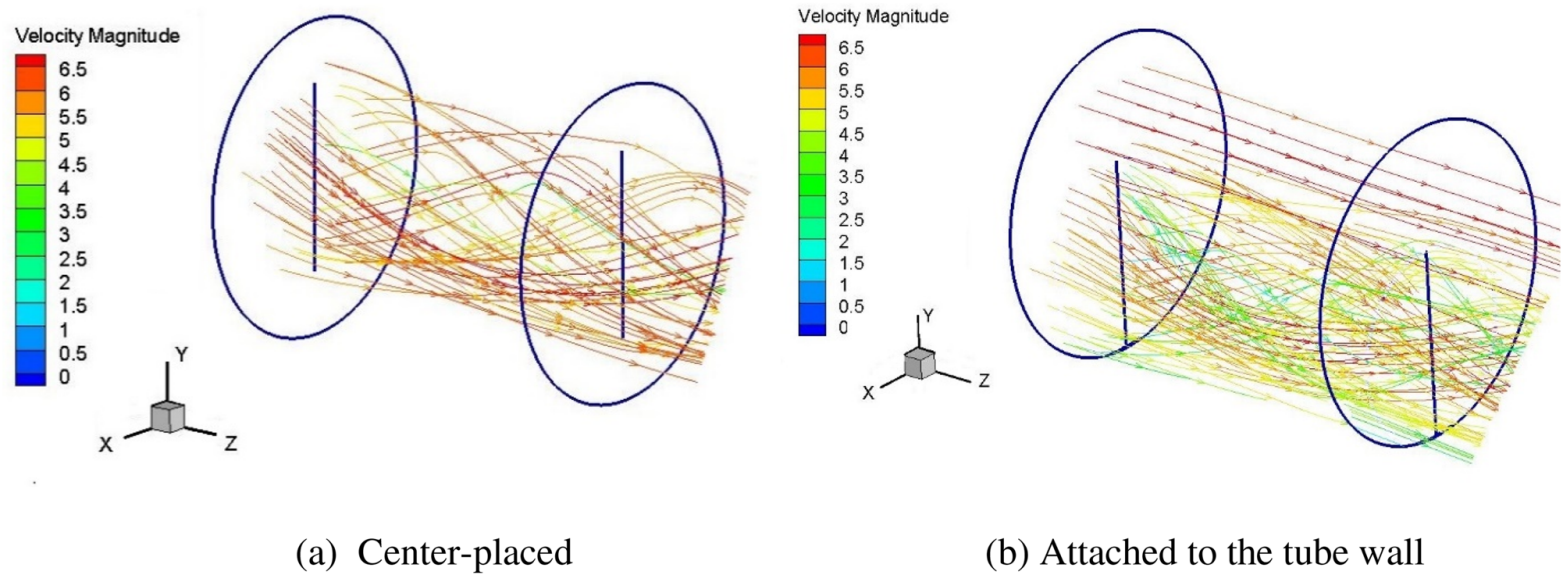
The stream line inside tube with twisted inserts at Re of 2600.

**Figure 6:**
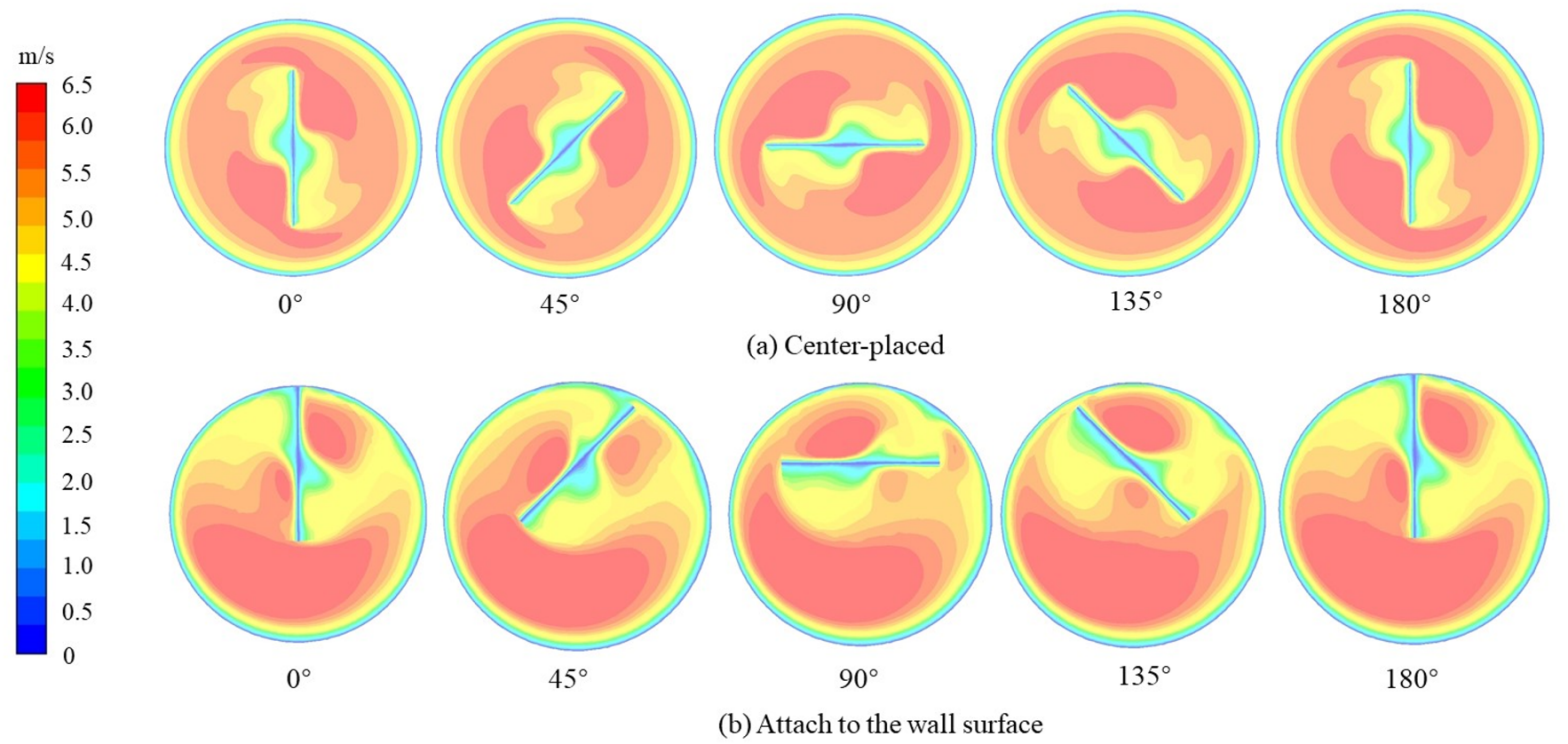
Comparison for velocity distribution in tubes.

Figure 5 shows the stream line inside the tube with twisted tape placed in the center and attached to the wall surface at Reynolds number of 2600 corresponding to the inlet velocity of 4 m/s. The width of the twisted tape is 12 mm. Cross sections were cut for the tube and twisted tape with a twist pitch of 24 mm, and the axial start position is 0.288 m from the inlet. Figure 6 presents five cross sectional velocity fields in the tube for the single twist pitch at various twisting angles: (a) for the center-placed case and (b) for the case that twisted tape is attached to the wall surface.

Compared to plain tubes, flow in the tube changes from linear motion to swirl flows around the twisted tape. For the tube with center-placed twisted tape, the swirl flows are generated in the center area of the tube, while the flow direction of the part of fluid near tube wall was not significantly changed, as shown in Fig. 5a. The swirling of the tape leads to a force (perpendicular to the tape surface) on the fluid close to the tape and accelerates the fluid, which results in higher velocities near the tube surface. Besides, the swirl flow contributes to fluid mixing. These two reasons are considered as main factors for the heat transfer enhancement induced by twisted tape inserts. When the twisted tape is attached to the wall surface, the swirl flows are generated near the tube wall, as shown in Fig. 5b. The fluid in the center region with higher velocity is transported to the near wall region by swirl flow, and the fluid with lower velocity in the near wall region close to the twisted tape is guided away from the boundary zone. The essential difference between these two placements is that the swirl flow occurs in high speed region or near wall region with a relative low speed.

As shown in Fig. 6a, high velocity zones are generated on both sides of the tape following the swirl direction. For example, at the twist angle of 0°, the high velocity zones occur at upper right area and lower left area which are in front of the swirl path of the tape. Low velocity zones are generated in the center of both sides of the twisted tape. The velocity distribution suggests that with the insert of twisted tape, part of fluid in the central region with higher velocity is swirled to be closer to the tube wall and low velocity region occurs at the center of the swirl. Besides, high velocity zones are also generated in the clearance between the twisted tape and the tube wall. Due to rotation symmetry, the flow field in the tube with center-placed twisted tape shows similar distribution at various twist angles.

The flow distribution in the tube with twisted tape attached to the wall surface is different with center-placed case. As shown in Fig. 6b, the flow distributions at different twist angles show different characteristics. When rotating the tape around its center axis, a circular shaped flow area will be formed, and we define it as the main swirl flow region. In the main swirl flow region, there generally exists two high velocity zones on each side of the tape following the swirl direction, though one might be weak than another. Besides, for the area out of the main swirl flow region, high velocity zones are generated in a wide area. Therefore, the swirl flow generated near tube wall promotes fluid mixing in the near wall region, generates a low velocity region in the center of the main swirl flow region on the tape, and forms a high velocity zone out of the main swirl flow region.

## Results and influence analysis of geometric parameters

**Figure 7:**
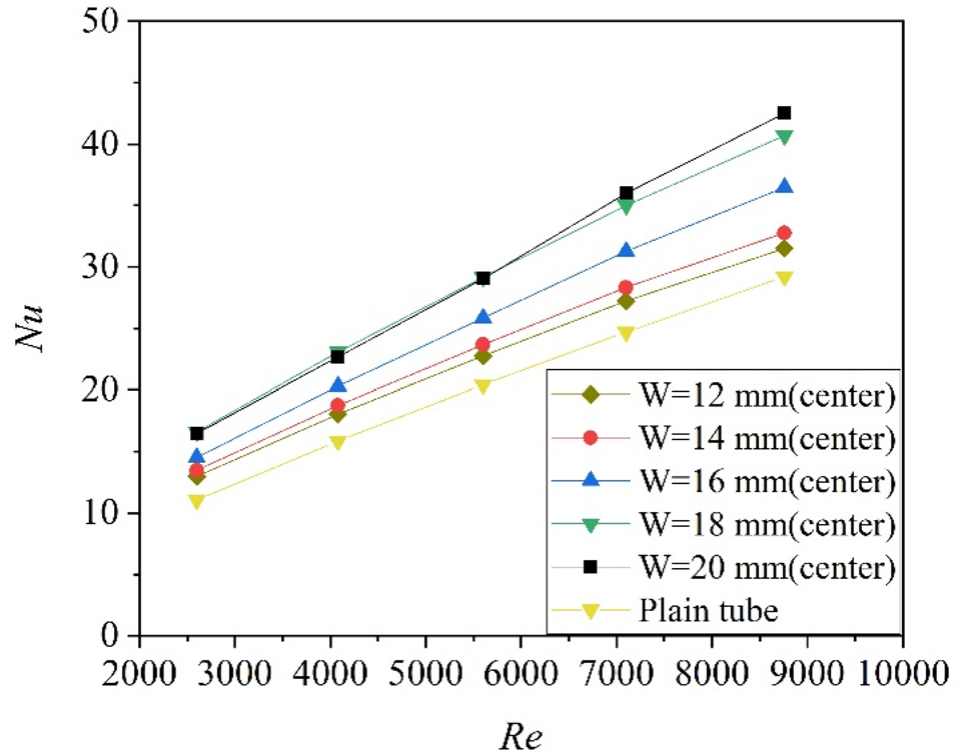
Nusselt numbers of tube with center-placed twisted tape inserts at different tape width.

The relationship curves between average Nusselt number and Reynolds number for the circular tube with center-plated twisted tape inserts at different tape width is shown in Fig. 7. Owing to the high velocity zones generated close to the near wall zone and the fluid mixing effect induced by the twisted tapes, all Nusselt numbers of the tube with center-placed twisted tape are higher than that of a smooth tube. Generally, the Nusselt numbers increases with the increase of tape width, though the values for width of 18 mm and 20 mm are very close at low Reynolds region. This may be caused by the thermal boundary layer disturb effect of the 18 mm case, for which the clearance between twisted tape and the tube wall is only 1 mm, smaller than the thermal boundary layer thickness. The Nusselt number of the tube fitted with twisted tapes at various width is 7–51% higher than the plain tube. Moreover, higher increase ratio of Nu was obtained at smaller Re.

**Figure 8:**
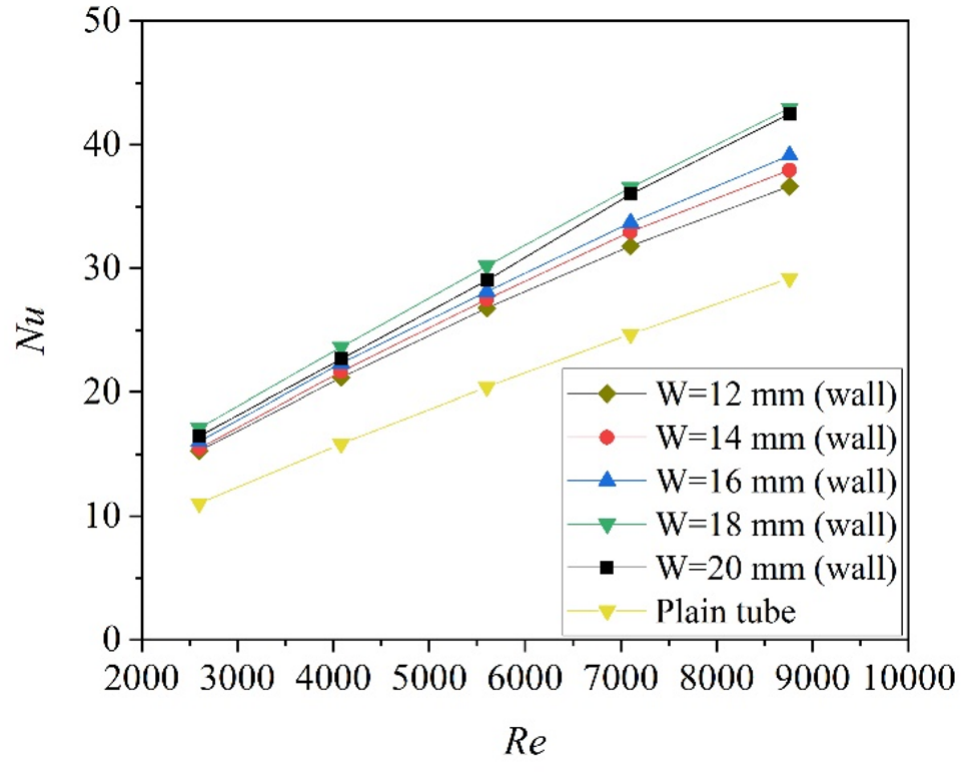
Nusselt numbers of tube with twisted tapes attached to wall surface at different tape width.

**Figure 9:**
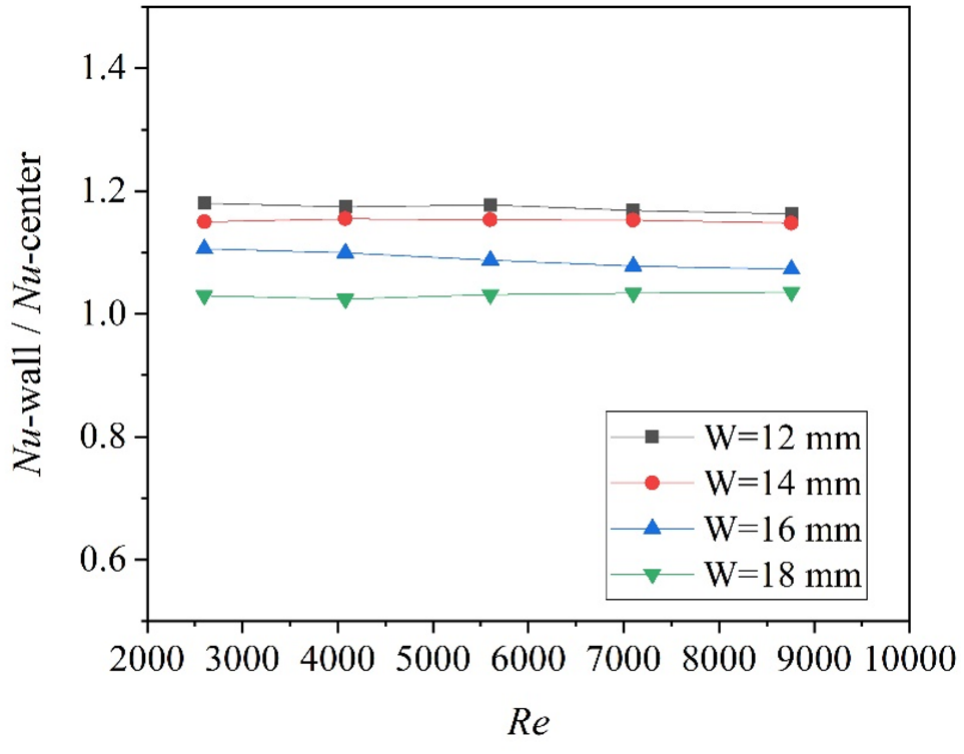
Variation of Nusselt number ratio (Nu-wall/Nu-center) with Re.

The Nusselt number for the circular tube with twisted tapes attached to the wall surface at different tape width is shown in Fig. 8. It can be found that the effect of width on Nusselt number is decreased in comparison to the center-placed case. The Nusselt number for the W = 18 mm case (attached to the wall) is larger than the W = 20 mm case. The ratio between the Nusselt number for the “attached to the wall surface” case (Nu-wall) and the Nusselt number for the center-placed case (Nu-center) is shown in Fig. 9. As can be seen that all the values of Nu-wall/Nu-center is larger than unity, which means the heat transfer for the tube with twisted tape attached to the wall is better than that for the tube with center-placed twisted tape. Furthermore, the smaller the width is, the higher the increasing ratio is obtained. The increasing ratio for Nusselt number ranged from 3 to 18%. Therefore, the heat transfer could be enhanced by off-center placement of the twisted tape.

## Performance evaluation

The performance of heat transfer enhancement of the twisted tape with different width is presented and the off-center placement effect is discussed. However, as we know that to obtain the enhancement of heat transfer by passive techniques usually leads to the increase of pump power for flow motion. In order to obtain a comprehensive evaluation of the heat transfer tube, the thermo-hydraulic performance parameter (ε) is employed, which is defined as follows^24^:

13 $${\varvec{\varepsilon}}=\frac{{\varvec{N}}{\varvec{u}}/{{\varvec{N}}{\varvec{u}}}_{0}}{{({\varvec{f}}/{{\varvec{f}}}_{0})}^{1/3}}$$

where, ${{\varvec{N}}{\varvec{u}}}_{0}$ is the Nusselt number of the plain tube, and ${{\varvec{f}}}_{0}$ is the friction factor of the plain tube.

**Figure 10:**
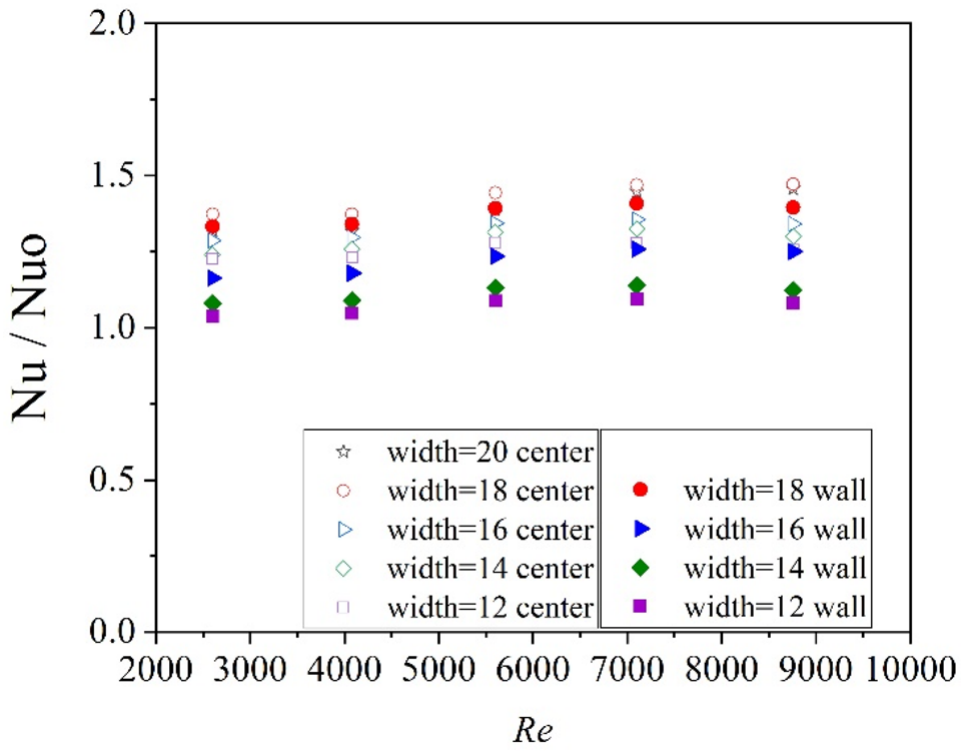
Nusselt number versus Reynolds number.

**Figure 11:**
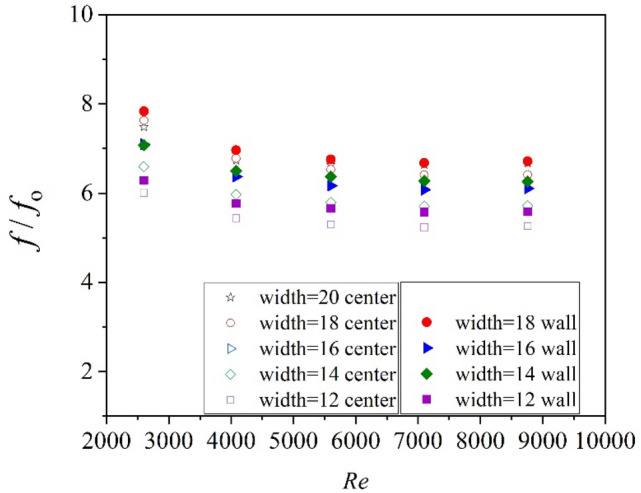
Friction factor versus Reynolds number.

**Figure 12:**
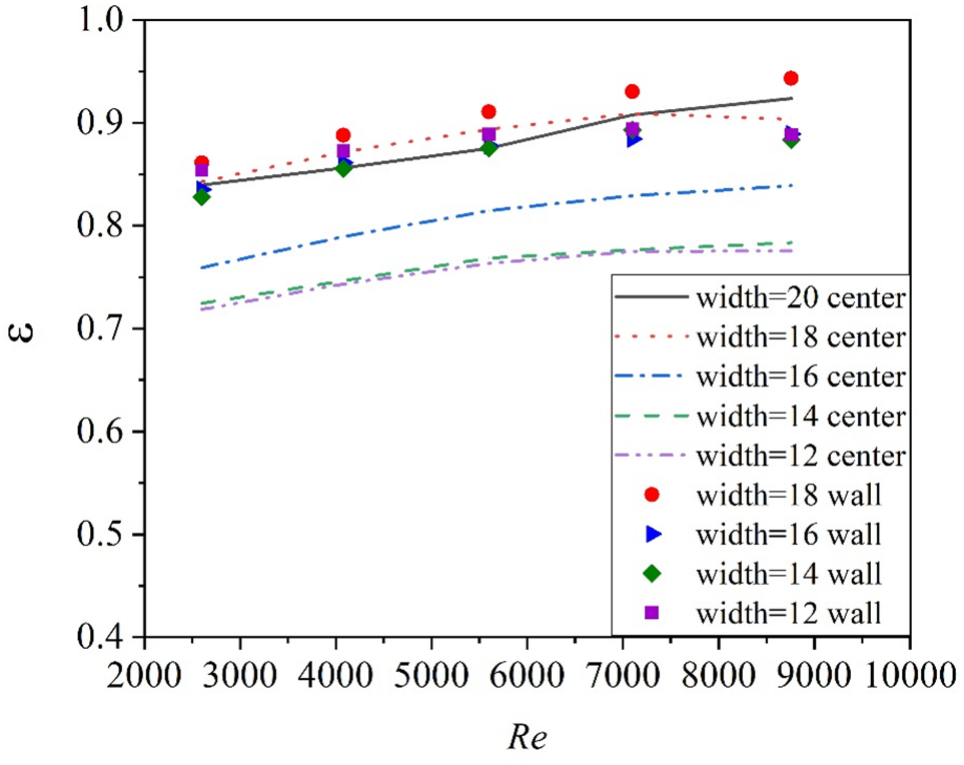
Thermal hydraulic performance factors for the circular tube with twisted tape.

The Nusselt number versus Reynolds number and friction factor versus Reynolds number are shown in Figs. 10 and 11. As can be seen, $Nu/{Nu}_{0}$ varies a little with Re, and the $f/{f}_{0}$ decreases with the increase of Re. Figure 12 shows the variation of the thermo-hydraulic performance parameter (ε) with Reynolds number for center-placed case and “attaching to the wall” case with different tape width. All the thermo-hydraulic performance parameter is below unity, which means the use of twisted tape inserts for convective heat transfer of helium gas flowing in a circular tube is not advantageous in energy saving in the calculated Reynolds region, though the heat transfer may be enhanced. The circular tube with twisted tape inserts attached to wall surface generally shows better performance than that with center-placed twisted tape.

## Conclusions

In the present study, the effect of off-center placement of twisted tape on flow distribution and heat transfer in a circular tube is discussed. The flow field, average Nusselt number, friction factor and thermo-hydraulic performance parameter of the tube are discussed and the effect of heat transfer enhancement induced by twisted tape inserts is analysed with Reynolds number ranged from 2600 to 8760.

The Nusselt number of the tube fitted with center-placed twisted tapes at various width is 7–51% higher than the plain tube and performance in low Reynolds region was found more effective than in high Reynolds region. The heat transfer for circular tube with twisted tape attached to the wall shows better performance than that for the tube with center-placed twisted tape. With a smaller tape width, a higher increasing ratio of Nu-wall/Nu-center is obtained. The increasing ratio for Nusselt number ranged from 3 to 18%.

The use of twisted tape inserts is not beneficial for energy saving. The thermo-hydraulic performance parameters for convective heat transfer of helium gas flowing in a circular tube are below unity for the calculated Reynolds region, which is mainly caused by the small density and heat capacity of gas when compared to liquid working fluids, such as water.

## Abbreviations

*c*_*p*_: Specific heat of helium gas (J/(kg K))
D: Diameter of the circular tube (m)
*f*: Friction factor of the tube
*g*: Gravitational acceleration (m/s^2^)
*H*: Twisted pitch (180º) (m)
*h*: Heat transfer coefficient (W/(m^2^ K))
k_f_: Thermal conductivity of helium gas (W/(m K))
L: Length of the tube (m)
*Nu*: Nusselt number, *Nu* = *D*/*λ*
*p*: Static pressure (Pa)
*Pr*: Prantl number
*q*: Heat flux (W/m^2^)
Re_*sw*_: Reynolds number based on swirl velocity, *U*_*s*_*L*_*s*_/*ν*
s: Twist ratio, s = H/W
*T*: Temperature (K)
*T*_*w*_: Wall temperature of the tube (K)
*T*_*out*_: Outlet temperature of the helium (K)
*t*: Time (s)
*u*_*c*_: Average inlet velocity (m/s)
*u*: *X* Component velocity (m/s)
*v*: *Y* Component velocity (m/s)
*W*: Width of twisted tape (m)
*w*: Z component velocity (m/s)
*Z*: Coordinate along the length of the tube (m)
α: Thermal diffusivity (m^2^/s)
*δ*: Plate (heater) thickness (m)
*ρ*: Density of helium gas (kg/m^3^)
*μ*: Molecular viscosity (kg/(m s))
